# SPISE for predicting the presence of atrial fibrillation in patients with coronary artery disease: a comparative analysis with TyG index

**DOI:** 10.3389/fendo.2026.1962511

**Published:** 2026-10-07

**Authors:** Meng-Wei Zhang, Dan Wang, Zheng Xiong, Chun-Mei Qi

**Affiliations:** Department of Cardiovascular Medicine, Second Affiliated Hospital of Xuzhou Medical University, Xuzhou, Jiangsu, China

**Keywords:** atrial fibrillation, coronary artery disease, insulin resistance, SPISE index, TyG index

## Abstract

**Objective:**

Insulin resistance (IR) acts as a common pathogenic mediator underlying both coronary heart disease (CHD) and atrial fibrillation (AF). The Single-Point Insulin Sensitivity Estimator (SPISE index) and triglyceride-glucose (TyG) index are two readily available laboratory surrogates for IR. This study sought to compare their independent predictive capacities for concomitant AF among patients with CHD.

**Methods:**

Baseline clinical data and laboratory measurements were collected to calculate SPISE index and the TyG index. All enrolled patients were stratified into AF and non-AF groups according to their baseline cardiac rhythm. Intergroup comparisons of the two metabolic biomarkers were conducted. Univariate and multivariate logistic regression models were constructed to identify independent correlates of AF. Restricted cubic spline (RCS) modeling was utilized to explore the nonlinear association between SPISE index and AF. Receiver operating characteristic (ROC) curve analysis was performed to quantify and compare the predictive capacity of SPISE index and the TyG index for AF. Predefined subgroup analyses were further carried out to evaluate potential effect modification across clinical strata.

**Results:**

SPISE index levels were significantly higher in the non-AF group relative to the AF group (*P* < 0.001). After comprehensive adjustment for all confounding covariates, SPISE index was independently inversely associated with prevalent AF (OR = 0.627, 95% CI: 0.528–0.746, *P* < 0.001). Restricted cubic spline combined with segmented regression analyses identified a significant nonlinear association between SPISE index and AF with a distinct inflection point. ROC curve analysis demonstrated that SPISE index exhibited superior predictive performance for AF risk compared with the TyG index (AUC: 0.646 vs. 0.566). The difference in AUC values between the two markers reached statistical significance according to Delong’s test. Stratified subgroup analyses revealed that the inverse correlation between SPISE index and prevalent AF remained stable across all predefined strata, including sex, age, smoking status, alcohol consumption, hypertension, diabetes mellitus, and BMI.

**Conclusion:**

SPISE index represents an independent correlate of prevalent AF in CHD patients. Compared with the TyG index, this convenient insulin sensitivity biomarker possesses superior screening value for identifying CHD individuals with concomitant AF.

## 1 Introduction

Atrial fibrillation (AF) is the most common sustained cardiac arrhythmia globally. It not only markedly deteriorates patients’quality of life but also substantially elevates the risks of major adverse cardiovascular events (MACE), such as ischemic stroke, heart failure, myocardial infarction and all-cause mortality (1). Over the past decades, the incidence and prevalence of AF have maintained a continuous rising trend worldwide, mainly attributable to population aging and the growing burden of hypertension, diabetes mellitus, obesity and other cardiovascular risk factors (2–4). It has been reported that roughly 40%-50% of patients with coronary heart disease (CHD) will develop AF during long-term follow-up. Accordingly, early identification of CHD patients at high risk of new-onset AF is essential for targeted risk factor management and better cardiovascular prognosis (5).

Insulin resistance (IR) is increasingly acknowledged as an upstream metabolic mediator of atrial arrhythmogenesis, independent of clinical hyperglycemia and established diabetes (6). IR triggers inflammatory activation of epicardial and pericoronary adipose tissue, boosts the secretion of pro-inflammatory cytokines including interleukin-6 (IL-6) and tumor necrosis factor-α (TNF-α), induces left atrial dilatation and myocardial interstitial fibrosis, and ultimately promotes the onset and progression of AF (7, 8). Though the hyperinsulinemic-euglycemic clamp remains the gold standard for quantifying IR, its cumbersome operation restricts its application in large clinical cohorts (9, 10). For this reason, simple serum-based surrogate markers have gained extensive popularity for routine IR assessment.

The triglyceride-glucose (TyG) index is the most widely applied surrogate of IR, calculated merely from fasting triglyceride (TG) and fasting glucose concentrations. A wealth of prior studies have validated its correlation with new-onset AF and post-ablation AF recurrence among CHD patients (11). The Single-Point Insulin Sensitivity Estimator (SPISE index) is a novel composite metric incorporating TG, high-density lipoprotein cholesterol (HDL-C) and body mass index (BMI) to reflect insulin sensitivity (12). Published meta-analyses have revealed that SPISE index exhibits superior predictive performance over HOMA-IR and the TG/HDL-C ratio for metabolic syndrome and cardiometabolic outcomes (13, 14). Nevertheless, existing research regarding SPISE index mainly centers on metabolic disorders including type 2 diabetes and non-alcoholic fatty liver disease (15, 16). To our knowledge, few investigations have explored the association between SPISE index and prevalent AF in CHD populations. Against this background, the present study aimed to compare the predictive efficacy of SPISE index and the TyG index for AF among patients with CHD.

## 2 Materials and methods

### 2.1 Study population

We retrospectively enrolled 538 inpatients diagnosed with CHD admitted to our hospital from 2022 to 2024. Inclusion criteria: Patients with definitive CHD diagnosis verified by clinical manifestations, laboratory tests and coronary imaging findings (17). Exclusion criteria: (1) rheumatic valvular heart disease; (2) thyroid dysfunction; (3) patients with prior artificial heart valve implantation; (4) history of cardiac radiofrequency ablation; (5) severe hepatic or renal dysfunction; (6) decompensated heart failure; (7) systemic inflammatory diseases and active infections requiring systemic treatment.

After screening, 23 patients were excluded, and a total of 515 eligible subjects were finally included in the statistical analysis, as illustrated in the flowchart in **Figure 1**.

**Figure 1:**
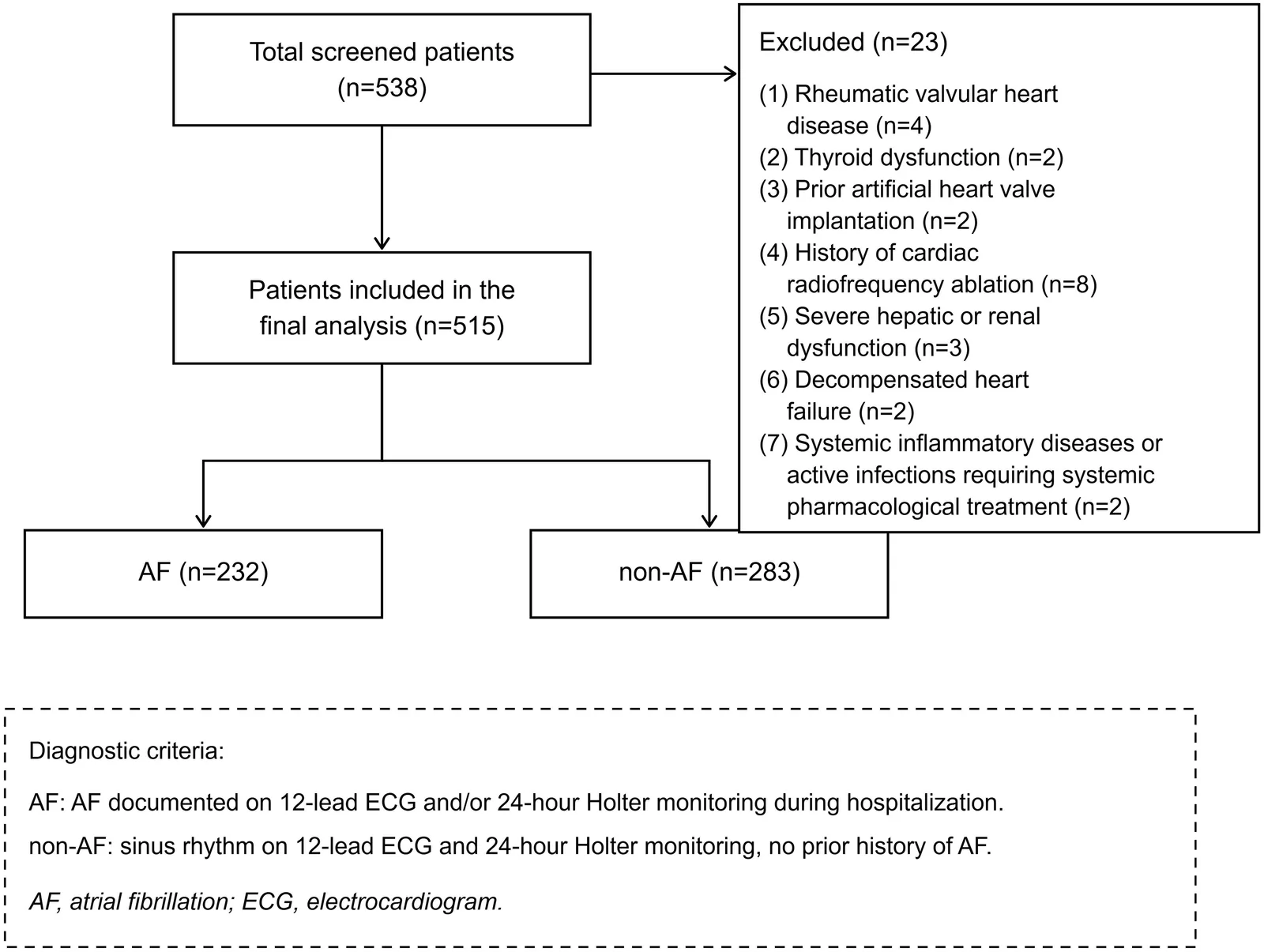
The flowchart of our research.

### 2.2 General information

Clinical data of all participants were extracted from the hospital electronic medical record system. Collected demographic variables covered age, sex and BMI. Documented comorbidities included hypertension and diabetes mellitus. Laboratory parameters consisted of fasting blood glucose (FBG), total cholesterol (TC), TG, HDL-C, low-density lipoprotein cholesterol (LDL-C), creatinine (Cr), uric acid (UA), white blood cell count (WBC), neutrophil count, lymphocyte count, monocyte count, platelets (PLT), IL-6 and TNF-α. All venous blood specimens were analyzed via standardized automatic biochemical analyzers and chemiluminescence immunoassays.

All participants received comprehensive transthoracic echocardiography performed on a Philips IE33 color Doppler ultrasound scanner. Recorded echocardiographic parameters contained left ventricular ejection fraction (LVEF), left atrial diameter (LAD), and left ventricular end-diastolic diameter (LVEDD).

### 2.3 Definition

BMI was calculated as body weight (kg) divided by the square of height (m).

SPISE index was calculated by the following formula:

SPISE index = 600 × HDL-C (mg/dL)^0.185^÷[TG (mg/dL)^0.2^ × BMI^1.338^]

The formula for the TyG index was shown as follows:

TyG index = ln [FPG (mg/dL) × TG (mg/dL) ÷ 2]

### 2.4 Follow-up

All participants received 1-year follow-up via outpatient visits or inpatient re-examinations. A total of 23 patients were lost to follow-up, resulting in a final analytical cohort of 515 subjects (295 males and 220 females). Among the included patients, 232 cases were diagnosed with AF based on the established diagnostic criteria (18): the disappearance of P waves on electrocardiography (ECG) or 24-hour Holter monitoring with replacement by irregular fibrillatory f waves; an atrial rate ranging from 350 to 600 beats per minute; completely irregular R-R intervals; normal QRS complex morphology; or a documented prior history of AF. Patients with concurrent CHD and AF were categorized as the AF group, whereas CHD patients without AF constituted the non-AF control group.

### 2.5 Statistical methods

All statistical analyses were conducted using SPSS version 25.0. Categorical variables were presented as counts (percentages), and between-group comparisons were performed using the χ² test. Normally distributed continuous variables were expressed as mean ± standard deviation, with intergroup comparisons conducted via the independent samples t-test. Non-normally distributed continuous variables were reported as median (25th percentile, 75th percentile), and between-group differences were compared using the Wilcoxon rank-sum test.

Multivariate logistic regression models were constructed to identify independent correlates of AF among patients with CHD. Receiver operating characteristic (ROC) curve analyses were performed to evaluate the predictive performance of biomarkers. The DeLong nonparametric test was utilized to compare the AUC values of paired ROC curves.

Three hierarchical regression models were established: Model 1 (unadjusted); Model 2 (adjusted for age and sex); Model 3 (further adjusted for age, sex, smoking status, alcohol consumption, hypertension, diabetes mellitus, FBG, Cr, UA, LVEF, and LAD). Restricted cubic spline (RCS) analyses based on the three regression models were applied to evaluate the nonlinear association between SPISE index and prevalent AF. Subgroup analyses stratified by sex, age, smoking status, alcohol consumption, hypertension, diabetes mellitus, and BMI were conducted to test the robustness of the SPISE index–AF association. A two-sided *P*-value < 0.05 was defined as statistically significant.

## 3 Results

### 3.1 Comparison of basic clinical characteristics between patients in the AF group and the non-AF group

Of the 515 patients with CHD, 232 had concomitant AF, yielding an overall AF prevalence of 45.05%. Significant intergroup differences were identified in age, BMI, heart rate (HR), FBG, HDL-C, Cr, UA, WBC, neutrophil count, monocyte count, LVEF, LAD, LVEDD, TyG index and SPISE index (all *P* < 0.05). Patients with AF demonstrated significantly higher TyG index values and lower SPISE index levels relative to non-AF participants. No significant between-group differences were detected in sex, hypertension, diabetes mellitus, alcohol consumption history, SBP, DBP, TC, TG, LDL-C and lymphocyte count (all *P* > 0.05). Detailed data are presented in **Table 1** and **Figure 2**.

**Table 1:** Comparison of baseline clinical characteristics between the AF group and non-AF group.

Variable	AF (n=232)	non-AF (n=283)	*P*-value
Clinical features			
Male/Female (n)	140/92	155/128	0.203
Age (years)	75 (70, 83)	68 (61, 74)	0.002
BMI (kg/m²)	24.80 (22.48, 27.34)	25.31 (23.15, 27.55)	<0.001
Hypertension, n (%)	163 (70.00)	197 (69.85)	0.873
Diabetes, n (%)	63 (26.52)	67 (23.76)	0.366
Smoking, n (%)	46 (20.00)	76 (26.95)	0.062
Drinking, n (%)	35 (14.35)	34 (12.06)	0.309
SBP (mmHg)	133 (120, 145)	132 (121, 150)	0.274
DBP (mmHg)	82 (74, 91)	81 (74, 90)	0.839
HR (per minute)	80 (70, 92)	75 (68, 84)	0.001
Laboratory values			
FBG (mmol/L)	5.48(4.79, 6.40)	5.17 (4.58, 6.16)	0.006
TC (mmol/L)	4.10 (3.18, 4.97)	4.09 (3.40, 5.14)	0.351
TG (mmol/L)	1.27 (0.96, 1.88)	1.22 (0.85, 1.72)	0.062
HDL-C (mmol/L)	1.05 (0.90, 1.26)	1.17 (0.97, 1.37)	0.000
LDL -C (mmol/L)	2.20 (1.64, 2.93)	2.23 (1.66, 2.95)	0.775
Cr (μmol/L)	70 (61, 84)	65 (56, 76)	<0.001
UA (μmol/L)	338(280, 410)	300 (257, 362)	<0.001
WBC (10^9^/L)	6.05 (5.00, 7.66)	5.73 (4.80, 6.76)	0.003
Neutrophils (10^9^/L)	3.69 (2.78, 4.77)	3.25 (2.57, 4.26)	0.003
Lymphocyte (10^9^/L)	1.50 (1.20, 2.11)	1.65 (1.31, 2.03)	0.187
Monocyte (10^9^/L)	0.50 (0.40, 0.62)	0.45 (0.37, 0.56)	0.001
PLT (10^9^/L)	194 (154, 232)	205 (168, 251)	0.011
IL-6 (pg/mL)	13.73 (6.00-24.43)	8.53 (4.53-15.69)	<0.001
TNF-α (pg/mL)	2.50 (1.50,2.72)	2.34 (1.32,2.54)	0.002
Echocardiography measurements			
LVEF (%)	56 (52, 59)	58 (57, 60)	<0.001
LAD (mm)	41 (39, 45)	37 (33, 41)	<0.001
LVEDD (mm)	48 (45, 51)	46 (43, 49)	<0.001
TyG index	8.65 (8.30,9.13)	8.57 (8.15, 8.96)	0.001
SPISE index	5.84 (4.96, 7.10)	6.60 (5.58, 8.27)	<0.001

AF, Atrial fibrillation; BMI, Body mass index; SBP, Systolic blood pressure; DBP, Diastolic blood pressure; HR, Heart rate; FBG, Fasting blood glucose; TC, Total cholesterol; TG, Triglycerides; HDL-C, High-density lipoprotein cholesterol; LDL-C, Low-density lipoprotein cholesterol; Cr, Creatinine; UA, Uric acid; WBC, White blood cell count; PLT, Platelets; IL-6, Interleukin-6; TNF-α, Tumor necrosis factor-α; LVEF, Left ventricular ejection fraction; LAD, Left atrial diameter; LVEDD, Left ventricular end-diastolic diameter; TyG, Triglyceride-glucose index; SPISE index, Single-point insulin sensitivity estimator.

**Figure 2:**
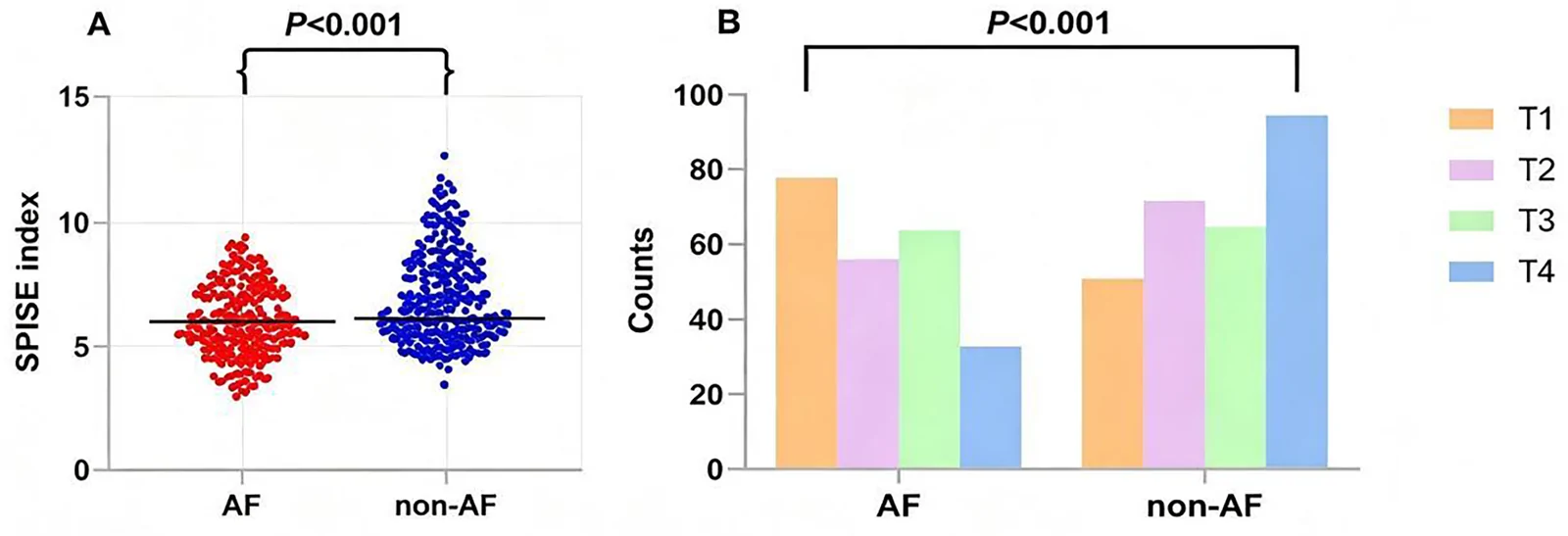
Comparison of SPISE index levels between the AF and non-AF groups **(A)**, and patient distribution across SPISE index quartiles (T1–T4) **(B)**.

### 3.2 Baseline characteristics across SPISE index subgroups

Significant intergroup differences in age, BMI, hypertension, diabetes mellitus, DBP, FBG, TC, TG, HDL-C, LDL-C, UA, WBC, neutrophil count, lymphocyte count, monocyte count, PLT, TyG index and AF prevalence were observed across SPISE index subgroups (all *P* < 0.05). Detailed data are presented in **Table 2**.

**Table 2:** Comparison of baseline clinical characteristics across SPISE index subgroups.

Variable	T1(n=129)	T2(n=129)	T3(n=129)	T4(n=128)	*P*-value
Clinical features					
Male/Female (n)	81/48	74/54	74/55	66/62	0.344
Age (years)	67 (58,73)	70 (65,78)	72 (66,80)	75 (69,84)	<0.001
BMI (kg/m²)	28.7 (27.3,30.1)	26.2 (25.3,27.6)	24.0 (22.9,25.0)	20.9 (20.0,22.1)	<0.001
Hypertension, n (%)	99 (76.74)	95 (73.64)	91 (70.54)	75 (58.59)	0.008
Diabetes, n (%)	39 (30.23)	29 (22.48)	42 (32.56)	20 (15.50)	0.007
Smoking, n (%)	38 (29.46)	31 (24.03)	28 (21.71)	25 (19.53)	0.273
Drinking, n (%)	22 (17.05)	20 (15.50)	12 (9.30)	15 (11.72)	0.240
SBP (mmHg)	132 (123,147)	135 (121,153)	132 (117,147)	130 (121,146)	0.378
DBP (mmHg)	83 (78,92)	84 (75,93)	79 (71,90)	80 (72,87)	0.001
HR (per minute)	78 (70,89)	78 (67,85)	78 (70,89)	76 (68,87)	0.616
Laboratory values					
FBG (mmol/L)	5.56 (4.81,6.74)	5.21 (4.71,6.15)	5.46 (4.77,6.48)	4.96 (4.47,5.86)	<0.001
TC (mmol/L)	4.29 (3.44,5.13)	4.40 (3.42,5.09)	4.16 (3.34,5.02)	3.74 (3.06,4.62)	0.015
TG (mmol/L)	2.00 (1.46,2.74)	1.36 (1.09,1.80)	1.12 (0.91,1.41)	0.84 (0.65,1.04)	<0.001
HDL-C (mmol/L)	0.98 (0.85,1.20)	1.07 (0.91,1.28)	1.19 (1.02,1.39)	1.25 (1.03,1.49)	<0.001
LDL -C (mmol/L)	2.34 (1.76,2.98)	2.47 (1.86, 3.06)	2.18 (1.73,2.86)	1.86 (1.37,2.60)	<0.001
Cr (μmol/L)	69 (60,77)	66 (56,78)	68 (58,77)	67 (58,84)	0.405
UA (μmol/L)	357 (288,417)	313 (259,397)	320 (269,369)	282 (247,357)	<0.001
WBC (10^9^/L)	6.46 (5.53,7.62)	5.88 (5.06,7.32)	5.71 (4.63,6.63)	5.66 (4.44,6.86)	<0.001
Neutrophils (10^9^/L)	3.83 (3.14,4.82)	3.48 (2.66,4.61)	3.17 (2.68,4.13)	3.25 (2.47,4.30)	0.001
Lymphocyte (10^9^/L)	1.70 (1.37,2.21)	1.65 (1.37,1.97)	1.59 (1.18,2.12)	1.38 (1.13,1.91)	<0.001
Monocyte (10^9^/L)	0.51 (0.41,0.64)	0.48 (0.38,0.59)	0.47 (0.37,0.58)	0.45 (0.35,0.56)	0.003
PLT (10^9^/L)	213 (181,289)	203 (172,244)	198 (159,233)	180 (152,227)	<0.001
IL-6 (pg/mL)	11.31 (5.17,20.28)	13.68 (5.19, 16.95)	11.24 (4.78,18.68)	11.73 (4.88,23.33)	0.967
TNF-α (pg/mL)	2.50 (1.66,3.40)	2.50 (1.58,3.18)	2.23 (1.47,2.75)	2.50 (1.59,3.11)	0.272
Echocardiography measurements					
LVEF (%)	57 (55,59)	58 (57,60)	58 (55,59)	57 (54,59)	0.128
LAD (mm)	40 (36,43)	40 (36,44)	39 (35,42)	39 (32,43)	0.294
LVEDD (mm)	47 (45,51)	47 (45,51)	47 (44,50)	46 (43,49)	0.042
TyG index	9.14 (8.71,9.57)	8.75 (8.45,8.99)	8.52 (8.23,8.89)	8.11 (7.88,8.43)	<0.001
AF	78 (60.47)	56 (43.41)	64 (49.61)	33 (25.78)	<0.001

AF, Atrial fibrillation; BMI, Body mass index; SBP, Systolic blood pressure; DBP, Diastolic blood pressure; HR, Heart rate; FBG, Fasting blood glucose; TC, Total cholesterol; TG, Triglycerides; HDL-C, High-density lipoprotein cholesterol; LDL-C, Low-density lipoprotein cholesterol; Cr, Creatinine; UA, Uric acid; WBC, White blood cell count; PLT, Platelets; IL-6, Interleukin-6; TNF-α, Tumor necrosis factor-α; LVEF, Left ventricular ejection fraction; LAD, Left atrial diameter; LVEDD, Left ventricular end-diastolic diameter; TyG, Triglyceride-glucose index; SPISE index, Single-point insulin sensitivity estimator.

### 3.3 Independent risk factors for prevalent AF in patients with CHD

Variables with P < 0.05 in the univariate logistic regression analysis were included in the multivariate regression models. After adjustment for multiple clinical, laboratory and echocardiographic confounding factors, SPISE index (OR = 0.627, 95% CI: 0.528–0.746, *P* < 0.001) and the TyG index (OR = 1.513, 95% CI: 1.003–2.283, *P* = 0.049) were identified as independent metabolic predictors of prevalent AF. Each 0.1-unit increment in SPISE index was associated with a 37.3% lower risk of AF, whereas each 0.1-unit increase in the TyG index corresponded to a 51.3% higher AF risk. Additional independent risk factors for AF comprised age, HR, UA, lymphocyte count, elevated IL-6 and TNF-α, reduced LVEF, and enlarged LAD (**Table 3**).

**Table 3:** Univariate and multivariate logistic regression analyses of risk factors for prevalent AF.

Variable	Univariate analysis	*P*-value	Multivariate analysis	*P*-value
	OR(95%CI)		OR(95%CI)	
Age (years)	1.023 (1.007–1.040)	0.006	1.046 (1.023–1.070)	<0.001
BMI (kg/m^2)^	1.175 (1.114-1.239)	<0.001		
HR (per minute)	1.022 (1.011–1.033)	<0.001	1.021 (1.008–1.034)	0.001
FBG (mmol/L)	1.078 (0.987–1.178)	0.096		
HDL-C (mmol/L)	0.362 (0.197–0.665)	0.001		
Cr(μmol/L)	1.019 (1.010–1.028)	<0.001		
UA(μmol/L)	1.004 (1.003–1.006)	<0.001	1.002 (1.000–1.005)	0.047
WBC (10^9^/L)	1.139 (1.044–1.242)	0.003		
Neutrophil (10^9^/L)	1.120 (1.015-1.236)	0.024		
Lymphocytes (10^9^/L)	0.926 (0.772–1.111)	0.409	0.823 (0.687-0.986)	0.034
Monocyte (10^9^/L)	3.776 (1.426-10.001)	0.007		
PLT (10^9^/L)	0.996 (0.993–0.999)	0.016		
IL-6 (pg/mL)	1.016 (1.001-1.030)	0.031	1.036 (1.014-1.058)	0.001
TNF-α (pg/mL)	0.781 (0.630-0.968)	0.024	0.752 (0.590-0.959)	0.022
LVEF (%)	0.920 (0.889–0.952)	<0.001	0.951 (0.913–0.990)	0.014
LAD (mm)	1.120 (1.084–1.158)	<0.001	1.087 (1.048–1.127)	<0.001
LVEDD (mm)	1.072 (1.033–1.112)	<0.001		
TyG index	1.575 (1.194-2.077)	0.001	1.513 (1.003-2.283)	0.049
SPISE index	0.693 (0.617–0.778)	<0.001	0.627 (0.528–0.746)	<0.001

AF, Atrial fibrillation; BMI, Body mass index; HR, Heart rate; FBG, Fasting blood glucose; HDL-C, High-density lipoprotein cholesterol; Cr, Creatinine; UA, Uric acid; WBC, White blood cell count; PLT, Platelets; IL-6, Interleukin-6; TNF-α, Tumor necrosis factor-α; LVEF, Left ventricular ejection fraction; LAD, Left atrial diameter; LVEDD, Left ventricular end-diastolic diameter; TyG, Triglyceride-glucose index; SPISE index, Single-point insulin sensitivity estimator.

### 3.4 Association between SPISE index and prevalent AF

Three sequentially adjusted multivariate binary logistic regression models were built to assess the association between SPISE index and prevalent AF. Model 1 represented the unadjusted model. Model 2 was adjusted for age and sex. Model 3 was further adjusted for the full set of covariates, including age, sex, smoking status, alcohol consumption, hypertension, diabetes mellitus, FBG, Cr, UA, LVEF, and LAD. After full-covariate adjustment, higher SPISE index was independently associated with reduced AF risk, indicating a negative correlation (OR = 0.606, 95% CI: 0.524–0.700, *P* < 0.001). SPISE index was analysed both as a continuous variable and a quartile-categorised variable (with T1 set as the reference group). Compared with participants in the T4 quartile, subjects in the T1 quartile exhibited a higher prevalence of AF (OR = 0.138, 95% CI: 0.073–0.262, *P* < 0.001). Detailed data are presented in **Table 4**.

**Table 4:** Logistics regression analysis model.

Group		Model 1	*P*-value	Model 2	*P*-value	Model 3	*P*-value
		OR (95%CI)		OR (95%CI)		OR (95%CI)	
SPISE Continuous	SPISE	0.693 (0.617-0.778)	<0.001	0.596 (0.520-0.683)	<0.001	0.613 (0.528-0.7120)	<0.001
SPISE categgorical							
	T1	–		–		–	
	T2	0.509 (0.310-0.836)	0.008	0.379 (0.224-0.642)	<0.001	0.441 (0.252-0.774)	0.004
	T3	0.644 (0.393-1.055)	0.080	0.451 (0.266-0.766)	0.003	0.568 (0.322-1.001)	0.020
	T4	0.227 (0.134-0.386)	<0.001	0.133 (0.073-0.242)	<0.001	0.144 (0.074-0.278)	<0.001

Model 1, unadjusted.

Model 2, adjusted for age, sex.

Model 3, adjusted for age, sex, smoking, alcohol consumption, hypertension, diabetes mellitus, fasting blood glucose, serum creatinine, uric acid, leftventricular ejection fraction, left atrial diameter.

95% CI, 95% confidence interval; OR, odds ratio; SPISE index, single-point insulin sensitivity estimator.

### 3.5 ROC curve comparative analysis of AF predictive efficacy

ROC curve analyses were conducted to compare the predictive performance of SPISE index and the TyG index for prevalent AF in patients with CHD, with the area under the curve (AUC) serving as the primary outcome metric (**Figure 3**). For individual-index prediction, SPISE index yielded an AUC of 0.646 (sensitivity = 66.08%, specificity = 85.78%, the optimal cut-off values =7.69, *P* < 0.001), whereas the TyG index had an AUC of 0.566 (sensitivity = 67.24%, specificity = 56.54%, the optimal cut-off values =8.44, *P* = 0.010). The DeLong test verified that the AUC of SPISE index was significantly higher than that of the TyG index (Z = 3.532; *P* < 0.001). After multivariable adjustment, the AUC for SPISE index reached 0.710 (95% CI: 0.669-0.749) in Model 2 and 0.792 (95% CI: 0.754-0.826) in Model 3. For the TyG index, the corresponding AUC values were 0.642 (95% CI: 0.599-0.683) and 0.775 (95% CI: 0.736-0.810), respectively. Across all adjusted models, SPISE index consistently exhibited better predictive performance than the TyG index. Compared with adding the TyG index to the baseline model, incorporation of SPISE index produced a larger improvement in discriminative ability, with an integrated discrimination improvement (IDI) of 0.077 (*P* < 0.001) and a continuous net reclassification improvement (NRI) of 0.451 (*P* < 0.001). Collectively, these results indicate that SPISE index provides superior incremental predictive value relative to the TyG index for identifying prevalent AF among patients with CHD.

**Figure 3:**
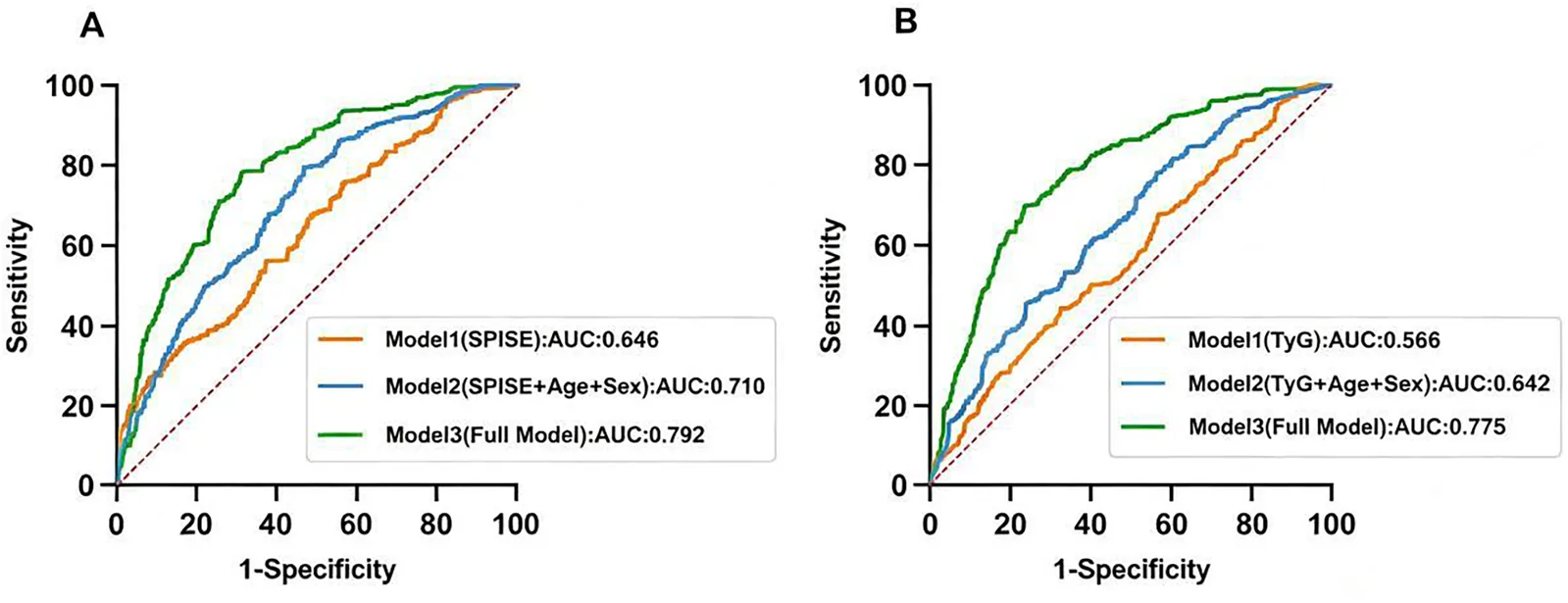
ROC curves for predicting prevalent AF in patients with CHD. **(A)** ROC curve of SPISE index for predicting prevalent AF; **(B)** ROC curve of the TyG index for predicting prevalent AF. ROC, receiver operating characteristic; AUC, area under the curve; SPISE index, single-point insulin sensitivity estimator; TyG, triglyceride-glucose index; CHD, coronary heart disease; AF, atrial fibrillation.

### 3.6 Restricted cubic spline analysis

After adjustment for potential confounders including age, sex, BMI, hypertension, diabetes mellitus, smoking status, and alcohol consumption, a significant nonlinear association between SPISE index and the odds of prevalent AF was identified (*P* for nonlinearity < 0.001). The odds ratio for AF gradually decreased with increasing SPISE index. Compared with participants at the median SPISE index level, individuals with lower SPISE index presented higher odds of prevalent AF, whereas higher SPISE index was associated with a lower risk of prevalent AF (**Figure 4**).

**Figure 4:**
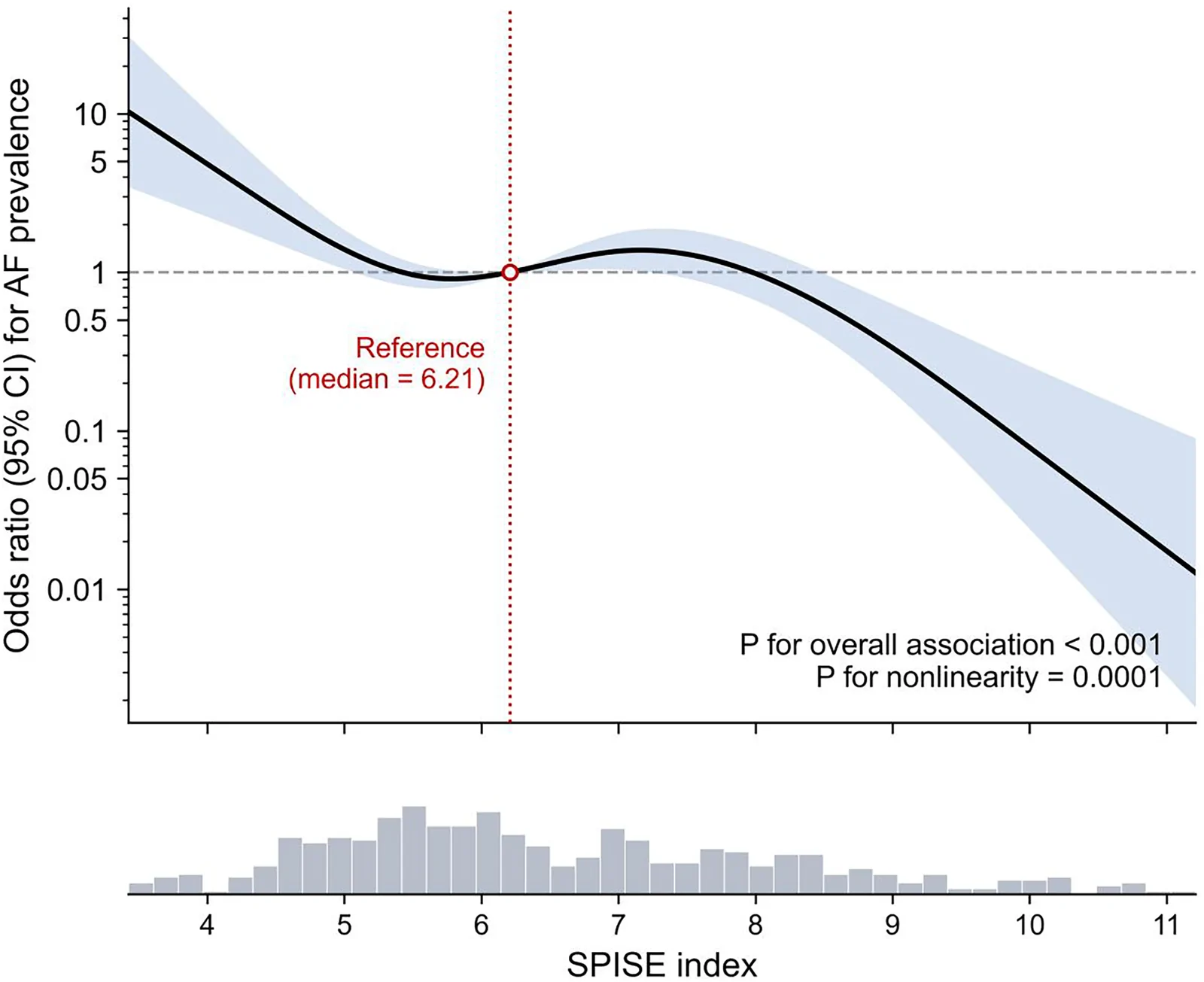
Restricted cubic spline analysis for the nonlinear association between SPISE index and prevalent AF in patients with CHD. AF, atrial fibrillation; SPISE index, single-point insulin sensitivity estimator.

### 3.7 Subgroup analysis

Subgroup analyses were performed to verify the robustness of the inverse association between SPISE index and prevalent AF. The protective effect of elevated SPISE index against AF remained stable and consistent across all stratified subgroups, including sex, age, smoking status, alcohol consumption, hypertension, and BMI (all *P* for interaction > 0.05). A marginally significant interactive effect was only detected for diabetes mellitus (*P* for interaction = 0.060), wherein the protective association of higher SPISE index with reduced AF risk was more prominent in CHD patients with diabetes mellitus (**Table 5**).

**Table 5:** Subgroup analysis of SPISE index with AF.

Variables	n (%)	OR (95%CI)	*P*	*P* for interaction	FDR-adjusted *P*
All patients	515 (100)	0.693 (0.617-0.778)	<0.001		
Sex				0.385	0.637
Female	220 (41.8)	0.653 (0.543-0.786)	<0.001		
Male	295 (47.5)	0.726 (0.625-0.844)	<0.001		
Age				0.285	0.637
≤ 71 years	269 (39.0)	0.681 (0.567-0.818)	<0.001		
> 71 years	246 (51.6)	0.592 (0.495-0.709)	<0.001		
Smoke				0.667	0.667
No	393 (47.3)	0.692 (0.606-0.790)	<0.001		
Yes	122 (37.7)	0.649 (0.498-0.845)	0.001		
Drink				0.173	0.606
No	466 (44.2)	0.718 (0.634-0.812)	<0.001		
Yes	69 (50.7)	0.545 (0.375-0.794)	0.002		
Diabetes				0.060	0.419
No	385 (43.9)	0.737 (0.648-0.838)	<0.001		
Yes	130 (48.5)	0.546 (0.410-0.726)	<0.001		
Hypertension				0.524	0.637
No	155 (44.5)	0.722 (0.596-0.874)	<0.001		
Yes	360 (45.3)	0.667 (0.574-0.775)	<0.001		
BMI				0.546	0.637
Normal (≤24)	198 (32.8)	0.621 (0.480-0.803)	<0.001		
Overweighht (>24)	317 (52.7)	0.691 (0.546-0.875)	0.002		

AF, Atrial fibrillation; BMI, Body Mass Index; SPISE index, single-point insulin sensitivity estimator.

## 4 Discussion

In this study, a total of 515 patients with CHD were enrolled. Multivariate logistic regression analyses demonstrated that SPISE index was independently and inversely associated with the risk of prevalent AF, whether treated as a continuous or categorical variable. This association remained stable and robust after comprehensive adjustment for multiple clinical, laboratory, and echocardiographic confounders. Consistent with our hypothesis, restricted cubic spline analyses further confirmed a significant nonlinear negative relationship between SPISE index and AF risk among CHD patients. Moreover, ROC curve and incremental predictive analyses indicated that SPISE index exhibited superior predictive accuracy for AF compared with the TyG index, as evidenced by higher AUC, IDI, and continuous NRI values. Collectively, these findings suggest that SPISE index may serve as a sensitive indicator of IR and a promising metabolic biomarker for the early identification of CHD patients at high risk of AF.

Accumulating prospective observational studies have demonstrated that IR is closely associated with the onset of multiple cardiovascular diseases, particularly AF (19, 20). The pathogenic link between IR and AF is primarily mediated by inflammatory responses and atrial structural and electrical remodelling. Specifically, IR drives the secretion of proinflammatory cytokines, including TNF-α and IL-6, which accelerate atrial fibrosis, disrupt normal atrial electrical activity, and increase arrhythmia susceptibility, ultimately facilitating the initiation and progression of AF (21–24).

The TyG index is a well-established and widely applied surrogate biomarker for IR and has been extensively validated in cardiovascular research (25–27). In contrast, SPISE index is a novel alternative IR indicator that comprehensively integrates BMI, TG, and HDL-C parameters and has shown promising correlations with multiple cardiovascular disorders (28, 29). Emerging clinical evidence has revealed the predictive value of SPISE index in AF prognosis; for instance, a study focusing on elderly patients undergoing AF radiofrequency ablation confirmed that SPISE index serves as an independent protective predictor of AF recurrence, with higher SPISE values corresponding to lower recurrence risks (30). Notably, clinical investigations exploring the association between SPISE index and prevalent AF in CHD populations remain limited, which highlights the novelty of the present study. Consistent with our findings, SPISE index exhibited superior predictive performance for AF compared with the conventional TyG index.

Our findings further validated and extended previous conclusions, demonstrating that SPISE index outperforms the TyG index in identifying CHD patients complicated with AF. After standardized adjustment for confounding variables within identical regression models, SPISE index consistently yielded superior predictive performance for AF relative to the TyG index. In the overall CHD cohort, higher SPISE was independently associated with a lower AF prevalence (OR = 0.693, 95% CI: 0.617-0.778, *P* < 0.001), further confirming its protective role against AF. To verify the robustness and heterogeneity of this association, we performed predefined subgroup analyses stratified by conventional cardiovascular risk factors. Interaction analyses revealed no significant effect modification by sex, age, smoking, alcohol consumption, hypertension, or BMI, with both raw and FDR-adjusted P values for interaction greater than 0.05, indicating that the protective effect of SPISE index against AF was stable and not affected by these traditional risk factors. Notably, a marginally interactive trend was observed only for diabetes mellitus (*P* for interaction = 0.060, FDR-adjusted *P* = 0.419). Stratified regression analyses further showed that the inverse SPISE-AF association was stronger in diabetic patients (OR = 0.546) than in non-diabetic patients (OR = 0.737), suggesting that the antiarrhythmic benefit of improved insulin sensitivity may be more prominent among CHD individuals with concomitant diabetes. These subgroup findings strengthen the clinical applicability of SPISE index. Unlike several metabolic biomarkers that only perform well in specific patient subsets, SPISE index maintains reliable predictive value for AF risk stratification across the entire CHD population. Combined with the evidence from multivariate logistic regression and ROC analyses that SPISE index outperforms the TyG index in predicting prevalent AF, supporting its potential as a promising metabolic biomarker for early AF risk screening and precise clinical intervention.

This advantage may stem from its comprehensive incorporation of anthropometric and lipid metabolic parameters, enabling a more holistic and accurate evaluation of systemic IR status, whereas the TyG index only relies on fasting triglyceride and glucose levels, which cannot fully reflect the complex metabolic dysregulation in CHD patients. Clinically, SPISE index provides robust incremental predictive value for AF risk stratification. Owing to its convenient calculation, non-invasive detection, and excellent discriminatory ability, SPISE index can be applied as a reliable auxiliary metabolic biomarker in routine clinical work to identify high-risk AF patients with CHD.

### 4.1 Limitations

Several limitations of the present study need to be addressed. First, this study was a single-center observational investigation that exclusively enrolled Chinese patients, which may restrict the generalizability and population representativeness of the results. The current findings are exploratory and lack external validation in multiethnic and Western cohorts, and standardized clinical cutoff values for SPISE have not yet been established. Future large-scale, multicenter, prospective studies with diverse ethnic populations are required to validate our conclusions. Second, relatively strict inclusion and exclusion criteria resulted in a highly selective cohort. The enrolled patients primarily represented a high-risk CHD population, rather than an unselected general population, which further limits the extrapolation of our findings to broader clinical settings. Third, all laboratory and metabolic indices were only measured at baseline. Dynamic changes in SPISE index during follow-up and their potential prognostic value for AF outcomes were not evaluated. Therefore, further well-designed prospective longitudinal studies are essential to confirm the causal association and optimize the clinical predictive efficacy of SPISE index.

## 5 Conclusion

A higher SPISE index is associated with a reduced risk of AF in patients with CHD. Compared with TyG index, the SPISE index has a higher predictive value. Therefore, SPISE index can be used as a simple and easily obtainable important clinical indicator for predicting AF, and it helps to promote the prevention and management of cardiovascular diseases, achieving more personalized strategies.

## Data Availability

The raw data supporting the conclusions of this article will be made available by the authors, without undue reservation.
